# Social security: individuals in socially reciprocal groups may perceive security from predators

**DOI:** 10.1093/beheco/araf008

**Published:** 2025-01-26

**Authors:** Conner S Philson, Clara Klassen, Kenta Uchida, Daniel T Blumstein

**Affiliations:** Rocky Mountain Biological Laboratory, P.O. Box 519, Crested Butte, CO 81224, United States; Natural Reserve System, University of California, Santa Barbara, Lagoon Rd. Building 520, CA 93106, United States; Centre for Research in Animal Behaviour, University of Exeter, Northcote House, Exeter EX4 4QG, United Kingdom; Rocky Mountain Biological Laboratory, P.O. Box 519, Crested Butte, CO 81224, United States; Graduate School of Agricultural and Life Sciences, University of Tokyo, 1 Chome-1-1 Yayoi, Bunkyo City, Tokyo 113-0032, Japan; Rocky Mountain Biological Laboratory, P.O. Box 519, Crested Butte, CO 81224, United States; Department of Ecology and Evolutionary Biology, University of California, 621 Young Drive South, Los Angeles, CA 90095-1606, United States

**Keywords:** antipredator behavior, flight initiation distance, group social structure, social network analysis, vigilance, yellow-bellied marmot

## Abstract

One of the most explored factors mediating antipredator behavior is group size, which generally predicts individuals in larger social groups allocate less time to antipredator vigilance while foraging. However, group size alone does not capture the full complexity of sociality. An individual’s ‘sense of security’, or their perceived risk of predation, is also influenced by an individual’s social connections. Further, group social structure – the pattern of all social interactions in a group – could explain additional variation in perceptions of security for the individuals that reside in the group. Using the time allocated to vigilance during foraging and flight initiation distance (FID) to quantify individuals’ social security, we explored whether individual yellow-bellied marmots (*Marmota flaviventer*) in tightly connected social groups looked less while foraging and had shorter FIDs. Using linear mixed effect models, we found modest support for the Social Security Hypothesis; individuals in more socially reciprocal groups may spend less time looking for predators while foraging. No measure of group social structure explained variation in FID. Measures of the immediate environment (the number of individuals within 10 m for vigilance and the distance from burrow and alert distance for FID) had effect sizes an order of magnitude greater than measures of social structure, suggesting an individual’s immediate environment has more of an impact on their antipredator behavior than the structure of their social group.

## Teaser

Animals perform and engage in a variety of behaviors to buffer predation risk. While more socially connected individuals living in larger groups generally perceive greater security from predators, we now show that individuals residing in more socially reciprocal groups also perceive greater security. This study was done as part of a long-term study of wild yellow-bellied marmots (Marmota flaviventer) at the Rocky Mountain Biological Laboratory.

## Introduction

By aggregating with conspecifics, prey may decrease their risk of predation through a variety of mechanisms. Previous studies have focused on describing antipredator behavior using group size effects and have shown that as group size increases, per capita risk of predation decreases. This phenomenon is known as the “Dilution Effect” (Cresswell 1994). The “Many Eyes Hypothesis” predicts that with the help of more eyes, ears, and noses to detect predators, larger groups can detect potential predation risks more quickly (Pulliam, 1973; Lima 1995). While group size is one of many attributes that may influence risk assessment (Hill and Lee 1998), group size alone does not capture the diversity and complexity of social relationships and patterns within groups. When individuals aggregate, they may engage in preferential relationships with one another. Thus, individuals in groups may vary in their connectedness with others. Additionally, groups vary in their overall connectivity and pattern of connection (Hinde 1976). Using social network analysis, we can quantify the number, frequency, and directionality (the initiators and recipients of interactions) of individual’s social relationships and their indirect social position within the group (eg who your direct social partners interact with) (Wey et al. 2008).

The Social Security Hypothesis predicts strong affiliative social relationships with conspecifics increase an individual’s perceived security (Mady and Blumstein 2017). For example, more socially isolated yellow-bellied marmots (*Marmota flaviventer*) cannot rely on conspecifics for their safety and thus alarm call more often (Fuong et al. 2015) and with higher call entropy (Fuong and Blumstein 2019). Marmots with weaker social relationships also flee at greater distances when approached by human observers (Szulanski et al. 2024). Impala (*Aepyceros melampus*) that are more central in their social network allocate less time to vigilance and spent more time foraging compared to their less-connected conspecifics (van Deventer and Shrader 2021). Additionally, crested macaques (*Macaca nigra*) exhibit a stronger response to playbacks of conspecifics’ alarm calls if they are produced by an individual with whom they share a strong social bond (Micheletta et al. 2012).

While individual sociality and social position may influence antipredator behaviors, the overall connectedness and structure of the social group may also influence the antipredator behaviors of each individual who resides in the group. Extending the Social Security Hypothesis to group social structure, residing in more connected and socially close groups may increase an individual’s perceived security, reducing perceived risk of predation. Group social structure has known fitness consequences for individuals within groups, from fish (Solomon-Lane et al. 2015), to mammals (Philson et al. 2022; Philson and Blumstein 2023a, 2023b) to insects (Costello et al. 2023). However, how group structure is associated individual perceptions of risk is still unknown. This is, in large part, because exploring emergent group social structure with individual-level risk perception requires many replicates of social groups and of marked individual’s antipredator behavior within those groups. Given the longitudinal and logistical demands, there are few study systems in the wild that meet these requirements. Because group social structure is pertinent to individual fitness, and group size has well established relationships with antipredator behavior, determining the roles of group social structure and social group size, relative to each other, is necessary to better understand individual antipredator behavior and social security in the wild.

To quantify antipredator behavior and risk assessment, the time an animal allocates to vigilance versus eating during spurts of foraging and flight initiation distance (FID) are commonly used, as seen in some mammals (Beauchamp et al. 2021), birds (Tätte et al. 2019), and insects (Shackleton et al. 2018). Animals detect perceived threats by allocating time to vigilance while foraging (Tyrrell and Fernández-Juricic 2015). Individuals who spend more time looking during foraging bouts can be perceived as more fearful and less secure. FID measures the distance at which a prey flees from an approaching threat (Ydenberg and Dill 1986). Therefore, FID reflects the individual’s risk assessment towards threats, such as an approaching predator. Many biological and environmental factors have been associated with FID, including body size (Weston et al. 2012), starting distance (Samia et al. 2013), vegetation conditions (Braimoh et al. 2018), the number of neighboring individuals (aggregation size) (Shuai et al. 2024), and individual social behavior (Szulanski et al. 2024). In summary, wildlife manages antipredator strategies via multiple behaviors to manage potential risks. Thus, further understanding what components of sociality, whether it be group size, individual social relationships, or group social structure, influence individual perceptions of risk is a pertinent question.

We asked how group social structure is associated with an individual’s ‘sense of security’ in a wild population of yellow-bellied marmots at and around the Rocky Mountain Biological Laboratory (RMBL) in Colorado where the population has been studied annually since 1962. Yellow-bellied marmots at the RMBL are a good system to test how social structure is associated with antipredator behavior because of the 20-yr dataset of individually marked animals with detailed social and antipredator behavioral data. Additionally, marmots have variation in their degree of antipredator behaviors (Armitage 2003) and group social structure (Blumstein 2013), and we know how their social structure relates to their individual fitness. Namely, group social structure has been related to dispersal (Schneidman et al. 2024), overwinter survival (Philson and Blumstein 2023a), mass gain rate (Philson et al. 2022), and reproductive success (Philson and Blumstein 2023b). Social network measures have been applied on an individual-level to study marmot social position and antipredator vigilance (Mady and Blumstein 2017; Szulanski et al. 2024). However, how group social structure relates to an individual’s sense of security has not been explored in this system, or others. With adult marmot FID repeatability being 0.132 (Blumstein et al. 2023), there is variation in FID that may be explained by group social structure.

Given the support for both group size effects for individual perceptions of security and the Social Security Hypothesis showing more social and connected individuals perceive greater security (Mady and Blumstein 2017; Szulanski et al. 2024), we extended the Social Security Hypothesis to the group-level to predict that individuals residing in social groups that are more tightly connected will allocate less time to antipredator vigilance when foraging. Namely, we predicted that individuals in more socially dense, close, connected, and reciprocal groups will perceive greater security by spending less time looking while foraging and having shorter FIDs whereas individuals in groups centrally structured around one or few individuals will perceive less security. Due to group size effects, we also predicted that individuals in larger social groups will perceive greater security. By fitting social group size with measures of group social structure in the same model, we will develop a more comprehensive and representative view of how group-level traits, including both size and structure, relate to antipredator behavior relative to each other in the wild.

## Materials and methods

### Study site and system

We studied yellow-bellied marmots in and around the Rocky Mountain Biological Laboratory (RMBL) in Gothic, Colorado, USA (38°77’N, 106°59’W; ca. 2900 m above sea level). Since 1962, the marmots have been under continuous study (Blumstein 2013; Armitage 2014), with detailed dyadic social interaction, FID, and vigilance data since 2003. Yellow-bellied marmots are a facultatively social mammal living in a matrilineal society which may include one or more adults, yearlings, and pups (Armitage 2014). Marmots are subject to aerial and terrestrial predation (Kelt and Van Vuren 2001) including, but not limited to, red-tailed hawks (*Buteo jamaicensis*), golden eagles (*Aguila chrysaetos*), coyotes (*Canis latrans*), red foxes (*Vulpes vulpes*), American badgers (*Taxidea taxus*), black bears (*Ursus americanus*), and mountain lions (*Puma concolor*).

Marmots at our study site hibernate for 7 to 8 mo of the year from mid-September to mid-April (Armitage 2014). During their active season, we trapped the marmots in walk-in Tomahawk traps and applied a set of unique ear-tags and unique dorsal fur mark with a nontoxic Nyanzol-D dye (Albanil Dyestuff Corp., Jersey City, NJ, USA) to distinguish individuals from afar. We use data from marmots studied annually at 8 colony sites spread 5 km apart along the bottom of the valley.

### Quantifying social behavior

Trained observers recorded social interactions between individual marmots from distances of 20 to 150 m using spotting scopes and binoculars. Behavioral data were collected at all 8 colony sites during the active season (mid-April to mid-September) during hours of peak marmot activity (0700 to 1100 h and 1600 to 1900 h) six days a week, weather permitting. Observers used all occurrence behavioral sampling to record all marmot social observations and interactions. Interactions were classified as affiliative (eg foraging together, grooming, and play behaviors) or agonistic (eg chasing, biting, or fighting behaviors) (full ethogram in: Blumstein et al. 2009). The initiator and recipient of the interactions were also recorded for the directionality of interactions. Affiliative social interactions comprised 88% of all observed interactions and 79% of interactions were between identified individuals (Philson and Blumstein 2023b).

### Social networks

We built social networks based on affiliative social interactions because the Social Security Hypothesis is based on affiliative, not agonistic, interactions. Networks included yearlings and adults and were constructed annually from 2003 to 2022. We filtered our data to include only social observations collected in April, May, and June because (1) vegetation was lowest at this time which allowed for accurate observations, (2) the most social interactions occurred during this time, and (3) yearlings had not yet dispersed (usually around late-June/early-July).

We defined social groups based on shared space use within the 8 colony sites (Pfau et al. 2023; Philson et al. 2024; Schneidman et al. 2024; Szulanski et al. 2024). To do so, we calculated simple-ratio pairwise association indices based on where individuals were trapped or seen within the same day using SOCPROG (Whitehead 2009). The resulting matrices were run through a community detection algorithm, MapEquation (Csardi and Nepusz 2006; Rosvall and Bergstrom 2008; Rosvall et al. 2009), to define social groups within colonies using the “igraph” package (Csardi and Nepusz 2006) in R (version 20.2.5; R Development Core Team 2023). Directed and weighted social networks were constructed from 42,369 affiliative social interactions between 752 unique individuals (with 549 being observed in multiple years) in 255 unique social groups-years across 20 yr (2003 to 2022).

For each unique social group, we calculated seven group-level social network measures to quantify social structure (Table 1) using “igraph.” Measures of social group connectivity included density, transitivity, cut points, and centrality. Measures of group homophily included reciprocity, inverse average path length, and degree assortativity. These seven measures were selected due to relevance across systems (Kasper and Voelkl 2009; Farine and Whitehead 2015; Sah et al. 2018), including in this marmot system (Philson et al. 2022; Philson and Blumstein 2023a, 2023b; Schneidman et al. 2024). These group-level traits are also generally extensions of individual-level social network measures used in past social security studies (Fuong et al. 2015; Blumstein et al. 2017; Mady and Blumstein 2017; Szulanski et al. 2024), facilitating comparison between individual and group studies of social security.

**Table 1.** Group-level social network measures, their descriptions, references, and predictions for their relationship with flight initiation distance (FID) and time allocated to vigilance.

Group measure	Description	Reference	FID	Vigilance
Density	The proportion of all possible social relationships in a group that are observed	Burt (1992); Wasserman and Faust (1994); Grund (2012)	**-**	**-**
Average Path Length^a^	How many social links individuals are from all others in the group	Watts (1998); Broder et al. (2002)	**-**	**-**
Cut Points^a^	A measure of social connectedness that quantifies how easily a group can fracture into two or more groups	Wasserman and Faust (1994); Borgatti (2006)	**-**	**-**
Transitivity	Quantifies group connectedness as the proportion of connected triads in the group	Wasserman and Faust (1994); Milo et al. (2002); Faust (2010)	**-**	**-**
Reciprocity	Measures the proportion of relationships within a group where both individuals initiate at least one interaction with each other	Wasserman and Faust (1994); ; Squartini et al. (2013)	**-**	**-**
Degree Assortativity	Tendency for social connections in a group to share similar a similar number of partners	Wasserman and Faust (1994); McPherson et al. (2001)	**-**	**-**
Centralization	Quantifies if interactions flow through few (high centralization) or many (low centralization) individuals in the group	Freeman et al. (1979); Wasserman and Faust (1994); Kang (2007)	**+**	**+**

^a^The inverse of average path length and cut points is presented for interpretability (so that as values increase, all measures can be interpreted as more connected).

### Antipredator vigilance

Trained observers conducted 2-min focal observations on individual marmots during bouts of foraging. The ethogram focused on head position and activity; vigilance was defined as the head being up while the individual likely looks for predators; foraging was defined when the head was down while an individual walks or ingests food (full ethogram in Blumstein et al. 2009). In addition to quantifying foraging, we also collected key contextual information: the colony location, number of individuals within 10 m (foraging aggregation size, incline of the slope, substrate (stones, talus, dirt, low vegetation, or high vegetation), and the distance the marmot was to the nearest burrow while foraging (Chmura et al. 2016). All focal recordings were scored in JWatcher 1.0 (Blumstein and Daniel 2004) to quantify the mean time each individual allocated to vigilance versus foraging during the two-minute foraging bouts recorded across the active season.

### Flight initiation distance and tolerance of approach

To quantify flight initiation distance (FID), a trained observer walked at a constant speed of 0.5 m/s directly towards a marmot that was not showing any alert behaviors at the time (Blumstein et al. 2004). We recorded the starting distance from the marmot to the observer, the alert distance from the marmot to the observer (when the marmot looked towards the observer but did not flee), and the flight initiation distance (when the marmot began to move away from the approaching observer) by dropping a flag for measurement after the animal fled and measuring with a measuring tape or laser-range finder (Yardagepro 400, Bushnell Performance Optics). Additionally, we also recorded the following environmental covariates to account for additional variation in FID (Stankowich and Blumstein 2005; Shuai et al. 2024): initial behavior, number of marmots within 10 m, slope of terrain and escape, substrate, trial number, and the marmot’s distance from a burrow during the trial. We did not measure FID when it was windy or rainy.

### Statistical methods

To explore the relationship between group social structure and the time individuals allocated to vigilance, we fit a linear mixed effects model using the package “lme4” (Bates et al. 2015). The following fixed effects were included in the model: number of individuals within 10 m, sex (female or male), age class (yearling or adult), social group size (ie social network size) and the seven social network measures. Year, colony, and marmot ID were included as random effects. We log-transformed cut points, degree assortativity, and centralization to meet model assumptions. All continuous fixed effects were then standardized (mean-centered across all samples). This model had a variance inflation favor (VIF) of 8.83 for density. A correlation matrix for all numeric fixed effects indicated that density and inverse average path length were highly correlated (0.88). We then removed inverse average path length from our model because density was more clearly connected to the Social Security Hypothesis. The model without average path length did not have multicollinearity issues and met all other model assumptions (Table 2). This model for vigilance had 2,625 total observations consisting of 529 unique individuals across 133 unique social groups in 20 yr. The vigilance measurements were recorded across the summer active season (mean ± SD = 12 June ± 23.3 d), with an average of 39.9 ± 15.8 individuals recorded each year an average of 3.13 ± 1.99 times each. Almost all (94.5%) of the vigilance recordings were conducted before 1 August. If an individual had multiple vigilance recordings per year, they still only had one social network value per social network measure for that year.

**Table 2.** Vigilance results. Estimates, standard errors, t-values, *P*-values, and marginal and conditional part R^2^ values for the fixed effects of the linear mixed models for vigilance.

	Estimate	SE	t-value	*P*-value	Marginal partial R^2^	Conditional partial R^2^
Intercept	0.360	0.021	17.019	**<0.001**	0.047 (0.037 to 0.075)	0.229 (0.159 to 0.328)
Social group size	−0.017	0.007	−2.402	**0.016**	0.005 (0 to 0.034)	0.186 (0.11 to 0.29)
Density	0.018	0.012	1.576	0.115	0.002 (0 to 0.032)	0.183 (0.107 to 0.288)
Transitivity	−0.003	0.008	−0.358	0.720	0 (0 to 0.03)	0.181 (0.105 to 0.286)
Reciprocity	−0.015	0.007	−2.222	**0.027**	0.001 (0 to 0.031)	0.182 (0.106 to 0.287)
Degree assortativity	−0.006	0.006	−1.020	0.308	0.002 (0 to 0.032)	0.183 (0.107 to 0.288)
Cut points^a^	0.009	0.006	1.557	0.120	0.003 (0 to 0.033)	0.184 (0.109 to 0.289)
Centralization^a^	−0.007	0.006	−1.247	0.213	0.002 (0 to 0.032)	0.183 (0.107 to 0.288)
Age class	−0.008	0.008	−1.081	0.280	0.001 (0 to 0.031)	0.182 (0.106 to 0.287)
Sex	−0.002	0.008	−0.253	0.801	0 (0 to 0.03)	0.181 (0.105 to 0.286)
Number w/in 10 m	−0.020	0.003	−5.891	**<0.001**	0.013 (0.004 to 0.042)	0.194 (0.119 to 0.297)

^a^Indicates log-transformation was done to better meet model assumptions.

For FID, we fitted a linear mixed effects model with alert distance, distance to burrow, number of individuals within 10 m, social group size, our seven social network measures, sex, age class, and trial number (to account for habituation) as fixed effects. We included alert distance as a fixed effect because of the strong positive correlation between FID and alert distance (Blumstein 2010; Cooper and Blumstein 2014) and because “best practice” suggests that AD should be included as a variable in FID models (Blumstein et al. 2015). We included year, colony, and marmot ID as random effects. To meet model assumptions, we log-transformed alert distance, distance to burrow, number of individuals within 10 m, group size, reciprocity, cut points, centralization, degree assortativity, and average path length. All continuous fixed effects were then standardized. This model had high VIF for density (11.37) and thus we again removed average path length due to a high correlation with density (0.93). The model without average path length did not have multicollinearity issues and met all other model assumptions (Table 3). This model for FID had 850 total observations between 299 unique individuals across 91 unique social groups in 16 years (FID data were not collected in four years due to logistical constraints). The FID measurements were conducted across the summer active season (mean ± SD = 29 June ± 19.7 d), with an average of 25.6 ± 11.4 individuals tested each year an average of 2.06 ± 1.23 times each. Almost all (93.4%) of the FID estimates were conducted before 1 August. If an individual had multiple FID recordings per year, they still only had one social network value per social network measure for that year.

**Table 3.** FID results. Estimates, standard errors, t-values, *P*-values, and marginal and conditional part R^2^ values for the fixed effects of the linear mixed models for FID.

	Estimate	SE	t-value	*P*-value	Marginal partial R^2^	Conditional partial R^2^
Intercept	3.126	0.109	28.690	**<0.001**	0.586 (0.52 to 0.651)	0.724 (0.676 to 0.767)
Alert distance	0.648	0.026	24.722	**<0.001**	0.444 (0.389 to 0.51)	0.581 (0.52 to 0.641)
Distance to burrow	0.155	0.022	7.022	**<0.001**	0.024 (0 to 0.124)	0.162 (0.046 to 0.27)
Number w/in 10 m	0.026	0.020	1.309	0.191	0.001 (0 to 0.102)	0.139 (0.02 to 0.249)
Social group size	0.030	0.053	0.561	0.575	0.001 (0 to 0.102)	0.138 (0.019 to 0.249)
Density	0.011	0.086	0.127	0.899	0 (0 to 0.102)	0.138 (0.019 to 0.248)
Transitivity	0.068	0.048	1.423	0.155	0 (0 to 0.1)	0.136 (0.017 to 0.247)
Reciprocity	−0.059	0.044	−1.340	0.182	0.004 (0 to 0.105)	0.142 (0.023 to 0.252)
Degree assortativity	−0.010	0.038	−0.269	0.788	0 (0 to 0.1)	0.136 (0.017 to 0.247)
Cut Points^a^	0.055	0.034	1.610	0.108	0 (0 to 0.1)	0.136 (0.016 to 0.246)
Centralization^a^	0.039	0.036	1.081	0.280	0 (0 to 0.102)	0.138 (0.019 to 0.248)
Age class	0.012	0.046	0.266	0.791	0 (0 to 0.102)	0.138 (0.019 to 0.249)
Sex	−0.064	0.050	−1.267	0.207	0.002 (0 to 0.104)	0.14 ( 0.021 to 0.25)
Trial number	−0.030	0.014	−2.240	**0.025**	0 (0 to 0.1)	0.136 (0.017 to 0.247)

^a^Indicates log-transformation was done to better meet model assumptions.

Using the “partR2” package (version 0.9.2; Stoffel et al. 2021) in R, we report marginal and conditional partial and semi-partial *R*^2^ values for each of our models. We then estimated 95% confidence intervals using 100 parametric bootstrap iterations.

## Results

For vigilance, we found that reciprocity (*B* = −0.015; *P* = 0.027) had a negative statistically significant relationship (ie individuals in more socially reciprocal groups spent less time looking for predators). No other measures of group social structure were statistically significant (Table 2). In addition to reciprocity, we found that the number of individuals within 10 m (*B* = −0.02; *P* < 0.001) and social group size (*B* = −0.017; *P* = 0.016) were negatively statistically significant with vigilance (ie individuals with more conspecifics nearby while foraging and in their social group spent less time looking around). This model explained 4.7% of the marginal variance and 22.9% of the conditional variance in vigilance. We found no statistically significant relationships between the measures of group social structure and FID (Table 3). We did find that alert distance (*B* = 0.648; *P* < 0.001), distance to burrow (*B* = 0.155; *P* < 0.001), and trial number (*B* = 0.03; *P* = 0. 025) were positively statistically significant with FID. This model explained 58.6% of the marginal variance in FID and 72.4% of the conditional variance.

## Discussion

Overall, we found very modest support for the extended group-level Social Security Hypothesis for vigilance, but no support at the group-level in our findings for FID. At the individual-level in this system, previous studies found that affiliative social relationships are associated with both FID and vigilance (Mady and Blumstein 2017; Szulanski et al. 2024). By exploring both vigilance and FID together, which reflect different aspects of antipredator behavior, we highlight the importance of examining multiple different antipredator behaviors to better understand the relationships with sociality.

For vigilance, the negative association with reciprocity may suggest that individuals residing in more egalitarian groups (in which individuals initiate interactions with one another more equally) allocate less time towards vigilance behavior and thus may perceive less risk than individuals residing in less socially reciprocal groups. In other mammalian systems, reciprocal interactions between individuals within a group are positively associated with cooperative antipredator response behaviors (Smith 1986; Wheeler 2008; Taborsky and Riebli 2020). For example, crested macaques (*Macaca nigra*) exabit a stronger response to playbacks of conspecifics’ alarm calls if they were produced by an individual with whom they share a strong social bond (Micheletta et al. 2012). Our modest finding that individuals in more socially reciprocal groups have higher perceived security may suggest that the value of social reciprocity for perceived safety scales from individuals to groups, and that this is possibly observed across taxa.

However, while reciprocity as a measure of group social structure had a statistically significant association with vigilance, it had a small effect size (*B* = −0.015; R^2^_m_ = 0.001), suggesting group reciprocity may have limited influence in the grand scheme of all factors that may influence individual vigilance. For example, we found that social group size (*B* = −0.017; R^2^_m_ = 0.005) and the number of individuals within 10 m of the focal individual (*B* = −0.02; R^2^_m_ = 0.013) had stronger statistically significant negative associations. Group size’s association supports the “Dilution Effect” (Cresswell 1994) while the number of conspecifics within 10 m suggests that foraging aggregation size and the immediate social surroundings of an individual may have a greater impact on antipredator risk assessment behavior than the more emergent group social structure.

To this same end, while no measures of group social structure explained variance in FID, three contextual fixed effects did: alert distance, distance from burrow, and trial number. The closer the observer was when the animal looked at the observer and the further an animal was from their burrow, the sooner the animal fled (as seen in Blumstein et al. 2005). This again suggests that the immediate surroundings and the environmental context that an individual experiences may have a larger influence on perceived security than social group structure when being approached by a threat. The negative association between FID and trial number suggests that repeated FID trials on marmots may have a modest habituation effect, with individuals fleeing at shorter distances with more trials. This has been shown in past studies in this system: animals subject to repeated trials of FIDs and exposure to human disturbance will tolerate closer human approach (Uchida and Blumstein 2021). This habituation to humans may contribute to the lack of a relationship with group social structure: individuals may rely on their immediate environmental context rather than social information given the known risk of humans from previous experiences.

Taken together, our results suggest that environmental and contextual factors have a stronger relationship with antipredator risk assessment and behavior than group social structure. How many individuals are nearby, how close the threat was when the animal alerts, and how many past FID trials have larger effect sizes (as measured by the semi-partial marginal part R^2^) and stronger relationships (as measured by the estimates) with antipredator escape behavior than any measure of group social structure (Tables 2 and 3).

Our results also suggest that FID and time allocated to vigilance measure two different components of antipredator behavior. The time an individual allocates to vigilance represents the response to the ambient risk in the environment during foraging (Lima and Bednekoff 1999; Armitage and Salsbury 2016), whereas the decision to flee reflects the response to more immediate threats, such as an approaching predator (Lima and Bednekoff 1999). Thus, our finding that group social structure relates to vigilance (the ambient risk) but not FID (the immediate risk) perhaps suggests that the structure of your group does not offer support or perceived security in a risky situation but does in a risky environment. This may align with the literature that suggests there are trade-offs and cost-benefit analyses underpinning risk-assessment behaviors in response to perceived predation risk (Lima and Dill 1990).

Contextualizing our results across taxa is impeded by the difficulty to generalize antipredator results across taxa. Prior studies have found that many environmental factors associated with FID are species-specific and context-dependent (Stankowich and Blumstein 2005). For example, aggregation size and repeated exposure to human disturbance (habituation) have been shown to have conflicting results with FID. Increased aggregation size may increase FID, supporting the “Many Eyes Hypothesis” (Morelli et al. 2019), and decrease FID, supporting the ‘Dilution Effect’ (Braimoh et al. 2018; Ardila-Villamizar et al. 2022). Both habituation and sensitization can occur in repeated trials of FIDs (Uchida and Blumstein 2021). For example, animals in urban areas can decrease their responsiveness to humans due to repeated exposure to harmless humans (Uchida et al. 2019) while human disturbance from increased hunting activity has increased the FID in wild reindeer (*Rangifer tarandus*) (Reimers et al. 2009). Despite the difficulties in generalizing across taxa, social reciprocity is observed across a wide range of taxa (Van Doorn and Taborsky 2012) and thus may influence antipredator behavior more broadly, at least for ambient risk behaviors. The more immediate social environment (ie number of conspecifics within 10 m) having a stronger relationship than the more passive and emergent group social structure may also be observed across taxa. However, both of these predictions require broader exploration across species and contexts.

In summary, we found that group social structure is only modestly associated with antipredator risk assessment and that group social structure is not associated with antipredator escape decisions when faced with an immediate and approaching threat. Overall, results suggest that environmental and contextual factors have a stronger relationship with antipredator risk assessment and behavior than group social structure. However, given group social structure is an important component of individual’s social environments across taxa and contexts (Solomon-Lane et al. 2015; Philson et al. 2022; Costello et al. 2023; Philson and Blumstein 2023a, 2023b), further exploration into how the structure of social groups may influence antipredator behavior is warranted.

## Data Availability

Analyses reported in this article can be reproduced using the data and code provided in Philson et al. (2025).
